# Second Clinical Report on Continued Fever, Based on an Analysis of Forty-Eight Cases

**Published:** 1851-12

**Authors:** Austin Flint

**Affiliations:** Prof. of the Principles and Practice of Medicine, and Clinical Medicine, in the University of Buffalo


ART. II.—*Second Clinical Report on Continued Fever, based on an analysis of forty-eight cases.* By AUSTIN FLINT, M. D., Prof. of the Principles and Practice of Medicine, and Clinical Medicine, in the University of Buffalo.

(Continued from Page 338.)

### SECTION ELEVENTH.

*Duration of the Disease. Circumstances attending Convalescence. Sequelæ. Mode of Dying. Fatality.*

*Duration.* In this, as in the first collection, the histories of several of the cases are defective in data for determining the time when the patients took to their beds, which, according to the rule I have pursued, fixes the commencement of the febrile career. In some cases the date of the first symptoms of the access were not ascertained. In these instances there is wanting the point of departure from which to measure the duration of the disease.

The period at which convalescence was pronounced, was determined by the ensemble of circumstances. According to my observations, it is not generally the case that a transition from the febrile career to convalescence takes place within the space of twenty-four hours. The change is usually not sudden. Occasionally it is so, but in the larger proportion of cases patients gradually advance to the point at which the febrile career may be declared to have ended, and the stage of convalescence entered on. Nor is it easy to fix on any arbitrary circumstances which shall suffice to mark the termination of the fever, as taking to the bed is considered to denote its beginning. The latter criterion is confessedly inaccurate, but any special event which may be selected for the former end would be still more open to objections. To make the fact of giving, for the first time, solid food as a criterion, after the plan of Louis, as remarked in the first Report, is to select an event which, of necessity, involves the pre-determination of the very point which it is made to indicate. The event simply denotes that at a particular day the patient was considered convalescent. It is plainly no evidence in itself of the fact of convalescence, but only of the opinion of the practitioner that the patient is convalescent. The patient assuredly may have the power to take solid food, if it be offered, while the disease is progressing. Moreover, it may not be considered unobjectionable by some, to give solid food prior to convalescence, and in such cases, the rule is not applicable.

There is, in fact, no alternative but to decide the point, in individual cases, by a fair and judicious estimate of all the symptoms; in other words, by an exercise of judgment based on the aggregate of circumstances. Such being the fact, a certain latitude follows. Different practitioners, it would be expected, might differ in the exercise of judgment in this particular; and the same practitioner might differ in applying his judgment in several cases. In short, it is by no means practicable in all cases to define the precise chronological limits of Continued fever with mathematical precision. This consideration, as it seems to me, disposes at once of the subject of critical days — a subject which has heretofore been much discussed. The question whether the disease ended on a critical, or non-critical day, is inapplicable to a large proportion of the cases of fever, inasmuch as the limits of the disease cannot be determined with exactness. This want of precision in fixing the duration, is not attended with any serious evils. For all practical purposes, sufficient accuracy is attainable.

The following tables will exhibit the facts pertaining to duration in so far as they are embraced in the histories. They will present an enumeration of the number of days from the commencement of the access, and from the time of taking to the bed to the time of convalescence, in all the cases that contain information on these points. They will embrace an enumeration of the number of days after admission into the hospital, to convalescence, and with respect to this point the data are, of course, in all cases available.

The number of days from convalescence to discharge, or the last date of record, and the whole number of days in hospital, will also be included. With respect to the latter point, however, I should premise that it is of but little practical importance, because other circumstances than those pertaining to the disease frequently determined the stay of patients in hospital. Some were anxious to get away as soon as possible, and it was an object with others to remain as long as possible, for reasons that were stated in the former Report.

TABLE EXHIBITING DURATION IN *TEN* CASES OF *TYPHUS.*

| | 1 | 2 | 3 | 4 | 5 | 6 | 7 | 8 | 9 | 10 | |
|---|---|---|---|---|---|---|---|---|---|---|---|
| No. of days from taking to bed to convalescence, - - - | 11 | | | 9 | | | 14 | 16 | 12 | 18 | Total, 80. Mean, 13 1-3. |
| No. of days from commencement of access to convalescence, - - | 15 | | | 14 | | | 14 | 20 | 15 | 21 | Total, 99. Mean, 16 1-2. |
| No. of days from admission into hospital to convalescence, - - | 11 | | | 8 | | | 14 | | 12 | | Total, 45. Mean, 11 1-4. |
| No. of days from convalescence to discharge, | 52 | | | 48 | | | 10 | | 27 | | Total, 137. Mean, 34 1-4. |
| No. of days in hospital, | 63 | | | 56 | | | 21* | | 39† | | Total, 182. Mean, 45 1-2. |
| *Fatal Cases.* | | | | | | | | | | | |
| From taking to bed, | | | | | 9 | 9 | | | | | Total, 18. Mean, 9. |
| From commencement of access, - - - | | | 21 | | 11 | | | | | | Total, 32. Mean, 16. |
| From admission into hospital, - - - | | 53 | 17 | | 9 | 9 | | | | | Total, 88. Mean, 22. |

* Sister of Charity connected with Hospital.

† Candidate for Sisterhood connected with Hospital.

TABLE EXHIBITING DURATION IN *TWENTY-NINE TYPHOID* CASES.

In comparing and commenting on the results contained in the foregoing tables, I will consider each division of the enumerations under a distinct head.

1. *Number of days from taking to the bed to convalescence.* The two types, *Typhus* and *Typhoid*, exhibit, in this aspect, the same mean duration within a fraction — being in the latter, 13, and in the former, 13 1-3 days. The fractional excess is in favor of the *Typhus* tpye. It is less in either type than in the first collection of hospital cases, in which the mean number of days, in the *Typhoid* cases, was 20; in the *Typhus* cases, 14 4-5. The first analysis, it will be seen, gave a marked disparity, in the two types, as respects this view of the duration — the number of days being greater in the *Typhoid* cases by nearly one-quarter. Thus, in comparing the results of the two analyses, there is a difference when the cases of both types, in the two collections, are brought into contrast, and when the two types are contrasted with each other in both collections.

The difference in the individual cases, in the duration after taking to the bed, is considerable. The *minimum*, in the *Typhoid* cases, is 8, and the *maximum*, 23 days; in the *Typhus* cases the variations in this respect are less, the *minimum* being 9, and the *maximum*, 18 days.

In the *fatal* cases the duration from the time of taking to the bed is noted but in *one Typhoid* case, and in that case was 41 days. It is noted in *two Typhus* cases, in each of which it was 9 days.

It is to be considered that the enumerations with respect to the duration from the time of taking to the bed, do not embrace all the cases in the present collection, as will be observed on looking at the tables. In some of the histories information on this point is not contained. The number of days is stated in *thirteen* (two fatal) of the *twenty-nine* cases of *Typhoid*, and in *eight* (two fatal) of the *ten* cases of *Typhus*. The same incompleteness existed in the tables embraced in the former Report. In the latter the number of days is stated in *six* of the *eighteen* hospital cases of *Typhoid*, and in *five* of the *twelve* hospital cases of *Typhus*.

1. *Number of days from commencement of the access to convalescence.* The mean number of days in the *Typhoid* cases, was 16 1-14 days. In the *Typhus* cases, it was 16 1-2 days. Here, again, the results in the two types are very nearly equal.

In the first Report enumerations were not made with respect to this point.

Of the *fatal* cases, the mean duration in those of the *Typhoid* type, was 24, and in *Typhus*, 16.

The enumerations of days under this head, contained in the tables, embrace *seventeen* (*three* fatal) of the *twenty-nine* cases of *Typhoid*, and *eight* (*two* fatal) of the *ten* cases of *Typhus*.

3. *Number of days from admission into hospital, to convalescence.* In the *Typhoid* cases, the mean number of days was 10 6-11. In the *Typhus* cases, it was 11 1-4. Here the disparity between the two types is not great. The *Typhus* type, it will be observed, exhibits the greater number of days.

In the first Report the mean number of days in the *Typhoid* cases, was 13 5-14; and in the *Typhus* cases, 11 10-11.

The cases in the first collection, thus, in both types, had a longer duration in this point of view, and as respects the relative duration of the two types, the results are the reverse of each other: in other words, the duration was longer in the *Typhoid* cases in the first, and longer the *Typhus* cases in the second collection.

In the *fatal* cases, the mean duration, dating from admission into hospital, in the *Typhoid* cases, was 19 1-3, and in the *Typhus* cases 22; the latter cases, as well as those which recovered, exhibiting a longer duration.

4. *Number of days from the date of convalescence to date of discharge.* In the *Typhoid* cases, the mean was 21 1-4 days. In the *Typhus* cases, it was 34 1 2 days. The disparity here was considerable. In this point of view, the duration, in the two collections, of both types, presents a striking contrast. In the first Report the mean duration of the *Typhoid* cases was 10 5 13, and of the *Typhus* cases, 16 3-7.

This difference is undoubtedly owing, in a great measure, to other circumstances than those pertaining to the condition of the patients consequent on the disease. Nearly all the patients in the last collection of cases were foreign emigrants supported by the State Emigrant Fund, which was continued until they were able to labor. Support of patients in this hospital from this fund, was not contributed prior to the year in which these cases were collected. Moreover, the pecuniary resources of the institution, which, prior to the past year had been quite limited, had become larger, so that patients who desired to remain until their health and strength became fully established, could be indulged in this respect, more than formerly. These considerations account for much, if not most of the prolonged stay in hospital after convalescence. The great disparity between the *maximum* and *minimum* periods of duration after convalescence, in individual cases, shows that the variations were due to extrinsic causes. Thus, in the *Typhoid* cases we find one patient remained only *two* days after the date of convalescence, another *four* days, and another *six* days; while, at the other extreme, one patient remained *forty-four* days, another *seventy* days, another *thirty-three*, etc.

The comparisons, as regards the whole number of days in hospital, are embraced under the foregoing heads, and therefore do not call for distinct consideration.

The duration of the disease dating from the time of taking to the bed, and from the time of admission into the hospital, is less than in the cases before analyzed. This is an interesting fact. How is it to be explained? It may be partially, or entirely due to a difference of habitude of this disease, in this particular, at different periods. That the disease does exhibit variations in duration, in different years, is shown by the observations of Dr. Jackson, of Boston. Dr. Jackson's Report on Typhoid Fever, contains a tabular exhibition of the average duration for each year of twelve successive years. This table presents considerable fluctuations, the extremes being nearly eighteen days in one year, and nearly twenty-six days in another. This is, in fact, the best explanation that can be offered. It may be supposed that the cases in the present collection were better managed, therapeutically, or hygienically, or both, and that the continuance of the disease was in that way abridged. There may be some force in this supposition, but it is impossible to say how much importance is to be attached to it. The difference in mortality was slight in the two collections, and not in favor of the last collection. Even admitting a marked improvement in the management of the disease to be susceptible of proof, it is not certain that the effect would be a notable diminution in the mean duration. It may be that such an effect would follow. The idea is certainly not irrational; but, in the present state of our knowledge, it is hypothetical.

The average duration of the disease, as developed by the present analysis, dating either from admission into the hospital, the time of taking to the bed, or the commencement of the access, is singularly short, being less than in any collection of cases, the results of the analysis of which have fallen under my notice.

Another point is important to be noticed. The average duration of the febrile career in cases of *Typhus*, has heretofore been found, by different observers, to be so constantly less than in cases of *Typhoid*, that this is considered to constitute one of the points of distinction between the two types. The results of the first analysis were in accordance with this rule. The present analysis, however, gives an average duration nearly equal, the slight difference telling against the rule. This suffices to show that as a law of the disease it will not be found invariably to hold good. This fact, however, is important to be remarked, disparity between the *Typhus* cases in the first and second collections, as respects duration, is not great; in other words, the cases of this type, in both collections, exhibit nearly the same average. That the average duration of the *Typhoid* cases is not greater than that of the cases of *Typhus* in the present collection is, thus, owing to the shorter duration of the former, not to an extended duration of the latter. This distinction should be borne in mind.

In connection with the above fact, it may be remarked, that the disparity of the results developed by the two analyses with reference, not only to that point, but in other particulars, illustrates the value of analyzing the cases collected in different years, separately, so as to bring the results into comparison with each other. In this way, the effects due to the eccentricities of the disease, or to the influences incident to different periods of time, are made to appear.

*Circumstances attending Convalescence.* In separating and arranging the facts contained in the histories, preliminary to instituting the enumerations and comparisons which it is the object of these reports to set forth, I omitted, inadvertently, in the present analysis, to direct particular attention to the circumstances preceding or connected with the occurrence of convalescence. As the first collection of cases were studied carefully with respect to this point, there does not seem to be an object sufficient to influce a re-examination of the cases of the present collection to supply the above omission. I have, therefore, nothing to add under this head to the conclusions submitted in the first Report.

*Sequelæ.* Aside from the facts which I shall present under the head of *relapses*, nothing which would properly be classed among the *sequelæ* of the disease came under notice save in one case. But it is to be considered that the patients were under observation only from the time of their admission, to their discharge. After leaving the hospital they were lost sight of, a large proportion being immigrants, *in transitu* to other places. My opportunities to study the probable influences of Continued Fever in the production of other diseases after a greater or less lapse of time, and its remote effects on the organism, have been quite limited; nor have I taken pains to record the few facts pertaining to this interesting subject which I might have collected.

In the single instance just referred to, the patient left the hospital on the thirty-third day after admission, and resumed the occupation of a laborer. Eighteen days subsequently he was again admitted with cough and febrile movement. He kept the bed for several days after admission, and was afterward about the ward, but remained feeble. On physical examination of the chest a month after the last admission, I found the signs of pulmo-tuberculosis. This was about the time my service ended, and the history from that date I have not learned.

*Relapses.* The facts contained in the histories of the cases relating to *relapses*, in other words, recurrence of febrile movement, with more or less of the symptoms belonging to the primary career of the fever, several days after convalescence was apparently established, exhibit a striking contrast with the results developed by the first analysis. In the former Report it is stated that "in none of the cases did *relapse* occur," and I added, "I have never witnessed what might properly be called a relapse after the career of Continued Fever was ended." From my observations up to the time of writ ng that Report, I was surprised at the statements made by some writers that patients who had passed through the career of Continued Fever were subject to relapses. While the cases which form the present collection were in progress, my attention was frequently called to the fact that during convalescence, and after patients had so far recovered as to sit up, and even walk about the ward, they were attacked with febrile movement, sometimes preceded by a chill, accompanied by anorexia, delirium. etc., these symptoms continuing for several days, when they again began to convalesce. In some instances I was disposed to attribute this recurrence of fever to imprudence in diet, exposure to cold, or over exertion, but it appeared to occur when no such cause could be assigned; and as respects the management of convalescence, the patients had the benefit of the same precautions and care as those whose histories were embraced in the first collection, and in the latter this sequence of the disease did not occur in a single instance. Moreover, the febrile movement, and associated symptoms, were out of proportion to those which might be expected to follow the imprudences just mentioned. The patients, in fact, appeared to pass through a second febrile career of short duration. This relapse of fever occurred in *fifteen* of the *forty-eight* cases. Of these *fifteen* cases, *nine* were of the *Typhoid* type, *one* was of the *Typhus* type, and *five* were cases of *Doubtful type.* I will present a summary of the recorded facts in these cases respectively.

### *Typhoid.*

Case 1. Convalescence was pronounced in this case on the ninth day after admission. Three days afterward there occurred recurrence of febrile movement, and delirium for two nights. The last record was made on the seventh day after the date of convalescence.

Case 2. The patient was distinctly convalescent on the ninth day after admission and taking to the bed, and the eleventh day from the date of illness. Seven days afterward she had recurrence of febrile movement, tongue became dry and hard, with anorexia, thirst, etc. The pulse rose to 128 and 144. In three days after the commencement of the relapse of fever, the symptoms exhibited marked improvement, and in ten days she was up and about the ward. She left the hospital twenty-seven days after admission.

Case 3. Convalescent on the sixth day after admission, on the seventh after taking to bed, and ten from the commencement of access. Four days after date of convalesceuce, a relapse of fever occurred, skin hot and dry, pulse 116, and at P. M., 128. The next day the symptoms exhibited improvement, and she was soon again convalescent. Left hospital twenty-seven days after admission.

Case 4. Convalescent on the eighth day after admission, and eleventh day after attack. Thirteen days after the date of convalescence, it is noted that he had had a slight relapse, but was then doing well. Left hospital twenty-nine days after admission.

Case 5. Convalescent on the tenth day after admission, and thirteen after date of attack. Five days after date of convalescence, a relapse of fever occurred, with anorexia, thirst, pain in head and limbs, congestion of face, pulse rising to 130. Continued five or six days. Remained in hospital twenty-three days.

Case 6. Convalescent on the tenth day after admission, and fifteenth after date of attack. Three days after the date of convalescence, had recurrence of febrile movement, epistaxis, etc., without any apparent cause, pulse rising to 128. The relapse of fever continued five or six days. Remained in hospital forty-one days.

Case 7. Convalescent on the eleventh day after admission, and date of taking to bed. Five days after date of convalescence attacked with chil pain in head, anorexia, thirst, dry and hot skin, pulse 104. The tongue became dry and hard. Pulse rose to 130. Again convalescent on fifth day after second attack. Remained in hospital forty-three days.

Case 8. Convalescent on the seventh day after admission, and eighth after date of attack. He was quite well, and walking about the ward, when, eight days after the date of convalescence, he took to the bed, with chill, pain in head and limbs, anorexia, pulse 116. Relapse of fever run about five days. Remained in hospital fifty-one days.

Case 9. Convalescent on the eighth day after taking to the bed, and twelfth after date of attack. Seven days after the date of convalescence, had return of febrile movement, and kept the bed for ten days. Remained in hospital forty-eight days.

*Typhus.*

Case 1. Convalescent on the eleventh day after admission, and taking to bed. Three days after date of convalescence, had return of febrile movement, which lasted for two days. Remained in hospital sixty-three days.

*Doubtful Type.*

Case 1. Convalescent on the seventh day after admission. Five days after the date of convalescence, attacked with pain in head, and in chest (substernal), febrile movement, etc. She kept her bed for ten days, when the symptoms again denoted convalescence. Remained in hospital forty-one days.

Case 2. Convalescent on the ninth day after admission, and taking to bed, and thirteenth after commencement of access. Five days after date of convalescence, relapse of fever occurred, with epistaxis, and he was confined to the bed eight days.

Case 3. Convalescent on the eleventh day after admission, and sixteenth from the commencement of access. Attacked on day of convalescence with chill, followed by febrile movement, etc. Relapse of fever continued five days. Remained in hospital thirty-four days.

Case 4. Convalescent on the twelfth day after taking to the bed, and thirteenth day after commencement of access. Four days after date of convalescence, relapse of fever occurred, pulse rising to 120. Four days afterward, again convalescent, pulse being the day before second convalescence, 104, and on the day of convalescence, 68.

Case 5. Convalescent on the eighth day after admission, the twelfth day after taking to bed, and fourteenth day after taking to bed, and fourteenth day after commencement of the access. Three days after the date of convalescence, relapse of fever occurred, which continued about eight days. During the second febrile career the parotid gland became somewhat swelled, which subsided in a few days, not proceeding to suppuration.

The occurrence of relapse in nearly one third of the whole number of cases, is one of the most interesting of the results of the present investigation. The cause, or causes, evidently pertained to the disease itself, not to extrinsic circumstances, for the latter, as has been stated, in so far as they were appreciable, were the same as had before existed when in not a single instance was this sequence noted. It is impossible even to conjecture what intrinsic conditions were involved in producing a special tendency thus incident to the year in which these cases were observed. The existence of such a tendency, however, is consistent with the well known fact that Continued Fever manifests, at different times, and places, certain peculiarities in the predominance of particular symptoms, the occurrence of complications, and in consecutive affections.

Relapses occurred, it will be perceived, in a larger proportion of cases of *Typhoid*, and those of *Doubtful type*, than of *Typhus*.

Another point is worthy of notice, viz., in all the cases characterized by this sequence, the disease finally ended in recovery. So far as these observations go, the occurrence of relapse of fever does not expose the patient to new dangers to life.

The inquiry may arise, whether convalescence was not prematurely pronounced in those cases in which relapses are stated to have occurred. Would it not have been more correct to have dated convalescence from the period when the so called relapse of fever terminated? To do so would, of course, do away with the occurrence of relapses. This inquiry suggests another fact in this section, viz., the remarkably short average duration in the cases in the present collection. This fact may seem to show that the career of the fever was not really ended in those cases in which convalescence was considered to be established, but in which febrile movement recurred after an interval of from two to five days. If the duration of the disease in these cases was estimated from the commencement to the date when convalescence was declared a second time, it would affect considerably the average, and bring it nearer that developed by the first analysis. According to this view, the peculiarity in the present collection of cases would consist in the frequent occurrence of a kind of pseudo-convalescence, or remission of febrile symptoms for several days. But, in opposition to this view, it is to be considered, that, in the cases referred to, the evidences of convalescence, at the time it was first pronounced, appeared to be unequivocal, and, in some instances, the patients sat up, and even walked about the ward before the relapse of fever became developed. Moreover, if the first were not a true convalescence, it would be expected that in the cases characterized by relapse of fever, the duration of the disease, prior to the first convalescence, would be in a notable degree less than the average period. I have computed the mean duration in these cases prior to the first convalescence, and find it to be 9 1-15 days. This is somewhat shorter than the average duration in all the cases, which was 10 6-11 for the *Typhoid* cases, and 11 1-4 for the cases of *Typhus*. The difference is not sufficiently great to militate much against the propriety of regarding what I have called the relapse of fever, as a recurrent febrile attack, rather than a continuance of the primary disease. With respect to this point, however, there may be room for difference of opinion.

NOTE. Since this Report was completed, my attention has been called to the views of Dr. Jenner, and some other British writers, on the subject of a distinct variety or species of fever, distinguished by the title of *Relapsing Fever*, characterized by a tendency to relapses, and other peculiarities. It may be a question whether the cases in the present collection in which relapses occurred, do not belong to a distinct type of Continued Fever. I propose to analyze these cases in order to ascertain if they are marked by any other distinctive traits than the occurrence of relapses. For the results of this analysis, and some remarks on the subject of Relapsing Fever, the reader is referred to the *Supplement* to the Reports.

*Mode of Dying.* The modes of dying, which in this, as in the first Report, will be considered to be resolvable either into asthenia or apnœa, are exhibited in the following tables:

*Seven Typhoid Cases.*

Case 1.—15th day after date of first record. Asthenia.
" 2.—5th day after admission. Apnœa.
" 3.—17th day after admission. (Perforation) Asthenia.
" 4.—4th day after admission. Not ascertained.
" 5.—9th day after admission. Asthenia.
" 6.—38th day after admission. (Peritonitis) Asthenia.
" 7.—2d day after admission. (Apoplectic coma) Apnœa.

*Four Typhus Cases.*

Case 1.—53d day (consecutive dysentery) after admission. Probably Asthenia.
" 2.—17th day after admission. (Pneumonitis set in on 12th day, when symptoms augured convalescence.) Apnœa.
" 3.—9th day after admission. Apnœa.
" 4.—9th day after admission. Apnœa.

*Fatality.* The number of cases that proved fatal, (eleven) has already been stated, and repeatedly referred to. The ratio is a fraction over twenty-two *per centum*. The percentage of fatality is somewhat greater than in the former collection of cases. In connection with this point, the remark made in the former Report is applicable, viz., the proportion of fatal cases would have been less, had not all cases in which it was supposed there was any room for doubt as to the diagnosis, been excluded. Some cases which were probably cases of Continued Fever were thus not embraced in the collection, and none of these were fatal cases. In a few instances, moreover, the histories were not recorded, or were found to be too incomplete to be available for the analysis. This also applied only to cases which recovered.

The ratio of fatality was considerably greater in the cases of *Typhus*, than in those of *Typhoid*, the proportion being as 4 – 10, or 2 – 5, is to 7 – 29, or a little less than one-quarter.

In the whole number of cases in both collections, the proportion of fatality is twenty-two *per centum*.

Adding together the cases of *Typhus* and *Typhoid*, respectively, in the two collections, and the result is, *fifty-nine* cases of *Typhoid*, and *twenty-three* cases of *Typhus*. The ratio of fatality to the whole number of cases of each type, thus added, is as follows: Of the *fifty-nine Typhoid* cases, *twelve* were fatal, i. e. a fraction over *twenty per centum*.

Of the *twenty-three* cases of *Typhus*, *eight* were fatal, i. e. a fraction less than *thirty-five per centum*.

These observations, thus, show in the two collections, separately and conjointly, a proportion of fatal cases considerably greater in the *Typhus*, than in the *Typhoid* type of Continued Fever.

The fatality from Continued Fever at different times, even when patients are placed in the same situation, and exposed to similar influences of hygiene, treatment, etc., has been found to undergo considerable fluctuations. This is shown in the Report of Dr. Jackson, of Boston, of the cases of *Typhoid* fever received at the Massachusetts General Hospital, for fourteen consecutive years. The variations in fatality during that period, from year to year, were between a *minimum* of a proportion of 1 to 3 1-2, and a *maximum* of a proportion of 1 to 25! Subsequently, at the same hospital, of *fifty-five* successive cases received from Nov. 1836 to Nov. 1838,* *not a single death occurred!* Louis states that a third of the patients, observed by him, affected with *Typhoid* fever, have died. These facts suffice to illustrate the striking contrasts as respects the rate of mortality that are presented in different years, contrasts which can only be accounted for by differences in the tendency to a fatal issue inherent in the disease at different periods. This feature in the history of Continued Fever, is not only interesting, but one which it is highly important should be borne in mind when we would estimate the effect of therapeutical, and other agencies, by the proportion of fatal cases.

---

* History, etc., of Fever, by Prof. Elisha Bartlett.

The deaths that occurred during the period in which the present collection of cases was made, were by no means equally distributed among the several months. Of the *eleven Typhoid* cases in the month of *October*, *four* were fatal. Of the *eight* cases in *November*, *all* recovered. Of the *eight* cases in *December*, *three* were fatal. The fatal *Typhus* cases were as follows: the only *two* cases that occurred in October, and *two* of *five* cases in March. *Two* cases which occurred in December, and *one* case in April recovered.

The number of cases although few, suffice to exemplify a greater tendency to a fatal result in some of the successive months of the same season than in others, a fact already developed by more extended observations.

### SECTION TWELFTH.

### *Examinations after Death.*

In the instances in which examinations after death were practicable, the objects were necessarily limited to the disclosure of characteristic lesions, in order to verify the diagnosis, and to exhibit them in connection with clinical instruction at the hospital. Want of leisure, and of proper facilities, precluded complete autopsical investigations embracing an inspection of all the important organs of the body. The examinations were generally confined to the small intestine, mesenteric glands and spleen, in some cases extending to other parts to which prominent symptoms during the progress of the disease were referable.

Examinations, limited as just stated, were made in *five*, of the *seven* fatal cases of *Typhoid*, and in *two* of the *four* fatal cases of *Typhus*.

The morbid appearances, so far as noted in the histories of the cases, respectively, are as follows:

### *Typhoid.*

Case 1. *Death on the fifteenth day after the date of the first record. Patient a porter in hospital, and had been in hospital for some time for chronic ulcer of the leg. Mode of dying by asthenia. Intellect clear a short time before death.*

*Abdomen.* The ilium presented a series of oval excavations situated in the Peyerian patches, the largest, near the cœcum, being about an inch and a-half in length, and three-fourths of an inch in width. The mucous membrane was destroyed over this, and several of the excavated spaces above, and their surfaces appeared to be covered with a very delicate transparent serous-like membrane, beneath which could be seen the fibres of the muscular tissue of the intestine. The Peyerian patches removed a few feet from the cœcum were thickened and softened, not ulcerated. Several of the solitary glands, si uated in the lower portion of the small intestine, presented the sites of ulcerations, or excavations, the surfaces covered with a delicate membrane.

Mesenteric glands enlarged. Peritoneal cavity contained about a pint of turbid serum.

Spleen enlarged and softened.

No note of other morbid appearances.

Case 2. *Death on the seventeenth day after admission. Perforation of intestine.*

No note of the appearances in this case was made until four weeks after the examination. It was omitted through inadvertence. The facts noted after the interval just mentioned, are the following: The ilium exhibited the characteristic lesions of Peyer's patches, the ulcerations being, for the most part, cicatrized. A perforation in one of the ulcerated spaces was discovered. The perforation, manifestly, was not from progressive ulceration through the coats, but from rupture, the point at which it existed being cicatrized.

Mesenteric glands enlarged.

Spleen enlarged and softened.

Evidences of peritoneal inflammation existed, viz., effused serum and fibrin.

Case 3. *Death on the morning of the fourth day after admission. Had been ill about ten days prior to admission. Active delirium existed before admission. Mode of dying not determinable by the history. Autopsy twelve hours after death.*

*Head.* Some subarachnoid effusion, and about an ounce of serum (estimated) in arachnoid cavity. Moderate congestion of vessels of brain. No effusion of fibrin, or opacity of arachnoid.

*Abdomen.* The ilium inspected for the space of about three feet above the cœcum. A large ulcerated patch of Peyer close to the cœcum, projecting from two to three lines above the plane of the mucous surface, and presenting a granular, jagged appearance. Above this point the Peyerian patches were salient, presenting an ulcerated surface, but, progressively, less and less in ascending the tube.

Numerous solitary glands projecting and ulcerated.

Mesenteric glands greatly enlarged—as large as filberts nigh to the cœcum.

Spleen enlarged, but did not appear to be abnormally softened.

Heart normal.

Case 4. *Death on the ninth day after admission, date of commencement of febrile career not ascertained. Mode of dying by asthenia, pulse*

*becoming extinct several hours before death, and mind apparently clear. Complicated with pneumonitis. Age of the patient about fifty years.*

*Head.* Not examined.

*Chest.* Lungs on left side presented deep engorgement. In the right chest the inferior lobe non-crepitant, œdematous, pitting on pressure; copious serous exudation on section, not spumous. About a pint of turbid serum contained within right chest. Slight fibrinous exudation on pleural surface of the solidified lobe. The remaining lobes crepitant, and not greatly congested.

*Abdomen.* Ilium presented Peyerian and solitary glands ulcerated, but in process of cicatrization, the surfaces being smooth, not granulated.

Affection of Peyerian glands gradually diminishing on ascending the tube.

Mesenteric glands moderately enlarged.

Spleen of about medium size, and softened.

Kidneys normal.

Liver presented no abnormal appearances.

Case 5. *Death, on the second morning after admission, in apoplectic coma. Had been ill four or five days before admission, but it is not stated whether confined to bed or not. Age of the patient thirty-eight.*

*Head*, first examined.

Considerable congestion of vessels of brain. Moderate increase of sub-arachnoid effusion. About an ounce of serum in arachnoid cavity.

*Chest.* Left lung firmly adherent with old pleuritic attachments. Considerable engorgement of lungs.

Heart normal.

*Abdomen.* Ilium contained thin yellowish matter. Peyer's patches prominent, and mucous tissue softened.

Mesenteric glands not enlarged. Solitary glands or lower part of ilium enlarged, giving the appearance of a papular eruption. Other portions of mucous tissue presenting nothing morbid.

Spleen enlarged and softened.

Kidneys enlarged, flabby, presenting some degree of granular degeneration.

*Typhus.*

Case 1. *Death on the seventeenth day after admission, and twenty-first after date of attack. Symptoms of pneumonitis on the twelfth day after admission. Mode of dying, by apnœa. Examination sixteen hours after death.*

*Chest.* Infetior lobe in left chest completely solidified. Pleuritic adhesions over this lobe. About half a pint of turbid serum in left pleural cavity.

Upper lobe free from disease. Right lung free from disease. Hypostatic congestion of lower lobe.

Heart presented no morbid appearances.

*Abdomen.* Ilium inspected for several feet above the cœcum. A single Peyerian patch nigh to the cœcum developed so as to be visible, presenting the *shaven beard* appearance. None other discoverable. The mucous surface through the whole space inspected, presented a series of arborescent vessels, the direction being transverse as respects the intestine.

Mesenteric glands not enlarged.

Spleen of about medium size, and softened.

Case 2. *Death on the ninth day after admission and date of taking to bed, and eleventh day from commencement of access. Mode of dying by apnœa.*

*Abdomen.* Peyerian patches developed so as to be visible.

Mesenteric glands very slightly enlarged near the cœcum.

Spleen not enlarged, nor softened.

Kidneys, liver, and heart, presented no morbid appearances.

As the correctness of the diagnosis is an important point, taken in connection with the intestinal appearances on the two fatal cases of *Typhus* in which examinations were made, I will give a succinct statement of the symptoms, in the history of each, which have an especial bearing on this point.

Case 1. In this case diarrhœa did not occur. Much of the time the abdomen was flaccid, but occasionally slight tympanites. Slight tenderness existed.

On the eighth day a faint maculated eruption was apparent. It was visible for three days. Its characters are not minutely described, but rose spots and maculæ are said to have been intermingled.

Epistaxis did not occur.

Muttering delirium was a prominent symptom, and became developed on second day after admission.

Capillary congestion of the surface was marked.

Case 2. Diarrhœa was not present in this case, nor tenderness, save that on one day he made complaint when firm pressure was made over the abdomen. Slight meteorism existed at first, but not afterward.

Delirium was a prominent symptom, consisting in incoherent muttering, and efforts to get out of bed.

The face presented deep congestive redness.

On the second day after admission and date of taking to bed, a copious dusky eruption appeared over the abdomen and chest, extending to the upper and lower extremities. The eruption was maculated, redness not disappearing on pressure. The eruption in this case was very copious, being almost confluent over the abdomen, and extended over the upper extremities even to the back of the hands.

